# Molecular Identification and Recombinant Expression of a Novel Antifungal Protein from Wheat-Associated *Paenibacillus polymyxa*

**DOI:** 10.3390/toxins18070318

**Published:** 2026-07-22

**Authors:** Xiaohong Ge, Zhikun Chen, Haoyuan Guo, Junjian Ran

**Affiliations:** 1 School of Food Science, Henan Institute of Science and Technology, Xinxiang Engineering Technology Research Center for Agricultural Products Processing, Research and Experimental Base for Traditional Specialty Meat Processing Techniques of the Ministry of Agriculture and Rural Affairs of the People’s Republic of China, Xinxiang 453003, China; gexh801210@163.com (X.G.); chenchien0711@163.com (Z.C.); 2 College of Food & Bioengineering, Henan University of Science and Technology, Luoyang 471023, China; ghaoyuan0711@126.com

**Keywords:** *Paenibacillus polymyxa*, purification, protein expression, antifungal activity, biocontrol

## Abstract

*Fusarium* head blight (FHB) caused by *Fusarium graminearum* leads to huge yield losses and mycotoxin contamination in wheat globally. *Paenibacillus polymyxa* with strong antagonistic activity was preliminarily identified. To clarify the key antifungal component, an extracellular protein was purified via ammonium sulfate precipitation, DEAE-52 anion-exchange and Sephadex G-75 gel filtration chromatography. SDS-PAGE showed a single band at 76 kDa. liquid chromatography–tandem mass spectrometry (LC-MS/MS) analysis confirmed this protein belongs to glycosyl hydrolase family with 86% sequence coverage. Biochemical characterization showed that the crude protein was stable at 40–90 °C and pH 3.0–9.0, sensitive to proteinase K, trypsin and neutral protease. The purified 76 kDa protein exhibited antifungal activity against *F. graminearum*. The gene encoding this protein was cloned and expressed in *Escherichia coli*. The renatured recombinant protein p76kd showed comparable antifungal activity to the native protein. This study purified and characterized a 76 kDa protein annotated as a glycosyl hydrolase via LC-MS/MS peptide matching; its antifungal function is presumed to originate from the conserved glycosyl hydrolase domain according to existing homologous research, which is distinct from previously reported lipopeptides or uncharacterized complexes. This protein provides a promising candidate for the biocontrol of FHB and related fungal diseases in cereal crops.

## 1. Introduction

*Fusarium* head blight (FHB) is one of the most destructive diseases affecting wheat, barley, and other small-grain cereals worldwide. To date, at least 17 *Fusarium* species have been associated with FHB, among which *Fusarium graminearum*, *F. avenaceum*, *F. poae*, *F. culmorum*, and *Microdochium nivale* are the primary causal agents, with the *F. graminearum* species complex being the most dominant and harmful pathogen [1,2]. FHB not only reduces grain yield and quality but also leads to the accumulation of mycotoxins such as deoxynivalenol (DON) and nivalenol (NIV), which pose serious threats to human and animal health [3,4]. The occurrence and severity of FHB are strongly affected by environmental conditions, agronomic practices, and their interactions [5,6]. No-tillage or reduced-tillage systems favor the survival of Fusarium species on crop residues, thereby increasing the risk of FHB epidemics.

Over recent decades, global demand for eco-friendly plant disease management has increased rapidly, driven by the need to reduce reliance on synthetic fungicides and mitigate environmental pollution and fungicide resistance. Biological control using beneficial microorganisms has emerged as a sustainable and safe alternative for controlling FHB [7,8]. Many bacterial and fungal antagonists have been reported to exhibit antagonistic activity against *F. graminearum*. Among these, *Bacillus* and *Paenibacillus* are particularly promising due to their ability to form endospores, tolerate harsh environments, and produce diverse antimicrobial metabolites [9,10].

*Paenibacillus polymyxa* is a well-documented plant growth-promoting rhizobacterium and biocontrol agent that can synthesize multiple antifungal compounds, including lipopeptides, hydrolases, and low-molecular-weight proteins [11,12,13]. Previous studies have shown that *P. polymyxa* strains produce various antifungal proteins and enzymes that inhibit the growth of phytopathogenic fungi such as *Pyricularia grisea*, *Rhizoctonia solani*, and *Fusarium* spp. [14,15]. Although strain was previously reported to show antifungal activity [16], the exact functional component had not been identified or heterologously expressed. Earlier studies only described crude extracts or mixtures rather than a purified protein.

In this study, a discrete 76 kDa glycosyl hydrolase protein from *P. polymyxa* was purified and identified, and prokaryotic expression for functional verification was performed. This 76 kDa glycosyl hydrolase exhibits unique structural and biochemical properties that have rarely been documented in previously characterized antifungal proteins derived from *Paenibacillus* spp.

## 2. Results

### 2.1. Localization of Antagonistic Activity

**Figure 1:**
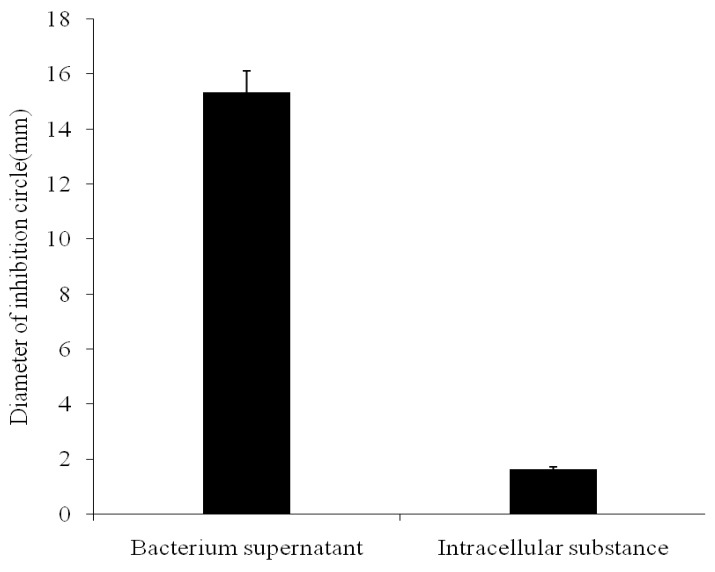
Comparison of antifungal activity between extracellular supernatant and intracellular fraction from *P. polymyxa*.

Following centrifugation of the fermentation broth of *P. polymyxa*, antifungal activity was separately quantified in cell-free supernatant, washed intact cells, and intracellular lysates. The cell-free supernatant displayed robust antifungal capacity with obvious inhibition zones, while negligible inhibitory effects were measured in intact cell pellets and intracellular extracts (Figure 1). This distinct discrepancy confirms that the primary antifungal effector molecule produced by strain is an extracellular secreted product. This distribution characteristic aligns with prior research on antagonistic proteins from model biocontrol bacterium *Bacillus subtilis*, whose active antifungal compounds are also largely released into the culture medium. This finding guided our subsequent purification workflow, as clarified cell-free supernatant was selected as the ideal initial sample to isolate the target antifungal protein and eliminate intracellular impurity interference.

### 2.2. Thermal, pH, and Protease Stability

A series of stability assays were carried out to characterize the antifungal activity derived from the culture supernatant of *P. polymyxa*, covering three experimental groups: thermal treatment, pH gradient adjustment and protease digestion treatment.

For thermal stability detection, aliquots of cell-free culture supernatant were subjected to incubation at a gradient of temperatures ranging from 40 °C to 90 °C, followed by agar well diffusion antifungal detection. Its antifungal activity gradually attenuates with temperature elevation, evidenced by a clear reduction in inhibition zone diameter from 16 mm (40 °C) to 6 mm (90 °C), yet detectable antagonistic activity remains at all tested temperatures, and all relevant measurement data were recorded and summarized in Table 1.

In the pH stability test, the pH value of fresh cell-free supernatant was adjusted to a continuous gradient ranging from 2.0 to 10.0 using dilute acid and alkaline solutions, and the antifungal activity of each pH-adjusted sample was determined separately. Obvious differences in antifungal performance were observed among samples with different pH values. The supernatant maintained intact antifungal activity under moderate and neutral pH environments. No detectable antifungal inhibition zones were observed for samples adjusted to pH values lower than 3.0 or higher than 9.0, while obvious inhibitory activity could be measured for all supernatant samples within the pH interval of 3.0–9.0, with all measurement results listed in Table 1.

To detect the protease sensitivity of the antifungal active substance, equal volumes of cell-free supernatant were individually treated with five different proteases, including proteinase K, pepsin, trypsin, neutral protease and papain, respectively, followed by antifungal activity detection after sufficient enzymatic reaction. Complete loss of antifungal inhibitory capacity was recorded in supernatant samples incubated with proteinase K, trypsin and neutral protease. By contrast, supernatant samples treated with pepsin and papain retained their original antifungal activity without visible attenuation, and all corresponding activity data were compiled in Table 1.

### 2.3. Purification and Molecular Weight Determination

Before attempting any further purification, an initial study to determine the appropriate pH was conducted. A 50 mM phosphate buffer at pH 6.8 was optimal, and the target protein retained approximately 90% antifungal activity after one month of storage at 4 °C.

**Figure 2:**
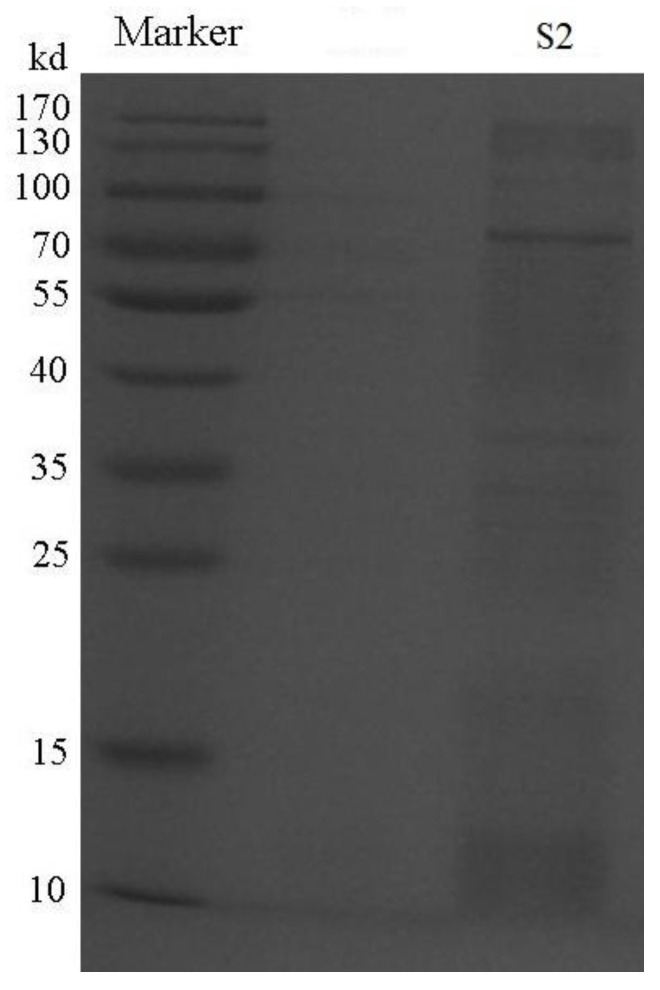
SDS-PAGE electrophoretic profile of purified 76 kDa antifungal protein.

Ammonium sulfate precipitation was employed as the initial purification step. Precipitates obtained at 60% saturation exhibited higher specific activity than those at other concentrations. In addition, 60% saturation yielded relatively less impurity precipitation compared with lower ammonium sulfate concentrations. The resulting precipitates were desalted via ultrafiltration and further subjected to DEAE-52 anion-exchange chromatography. Most of the active component bound tightly to the DEAE-52 column via anionic interactions. Five fractions (D1-D5) were collected and their antifungal activities were evaluated. Fraction D4 displayed significantly higher antifungal activity than the other fractions; however, it had already been reported [16]. Fraction D3 also displayed significantly higher antifungal activity (Table 2). The active fraction D3 was further purified using Sephadex G-75 gel filtration chromatography and separated into three subfractions, S1, S2 and S3, at a flow rate of 0.1 mL min^−1^. Fraction S2 exhibited strong antifungal activity, whereas S1 and S3 showed almost no activity (Table 2). The final specific activity of fraction S2 was determined to be 120 U g^−1^, representing a 1600-fold purification relative to the crude extract. SDS-PAGE followed by Coomassie Blue R-250 staining revealed that fraction S2 displayed a single distinct band with a molecular mass of approximately 76 kDa (Figure 2).

Combined data of activity localization, stability characterization, chromatographic separation and recombinant protein functional reconstitution collectively demonstrate that this purified 76 kDa glycosyl hydrolase protein acts as the primary secreted antifungal effector of *P. polymyxa*, while small-molecule metabolites and bacteriocins contribute minimally to the strain’s antifungal capacity. This study characterized the principal component responsible for its anti-Fusarium activity.

### 2.4. Identification by LC-MS/MS

The distinct protein band corresponding to a molecular weight of 76 kDa was manually excised from the polyacrylamide gel after SDS-PAGE separation. The excised gel slice was fully rinsed to remove residual electrophoresis buffer and staining reagents, followed by in-gel digestion using trypsin to generate short peptide fragments. The digested peptide mixture was subsequently subjected to MALDI-TOF-TOF tandem mass spectrometry to acquire peptide mass fingerprinting and fragment ion spectral data. All acquired mass spectral data were matched against the protein reference database of *P*. *polymyxa* deposited in the NCBI database for homologous sequence alignment. The database search retrieved four candidate protein sequences that shared high amino acid homology with the peptide fragments obtained from the 76 kDa target band. Ranking by matching score, the two highest-confidence candidate proteins contained a glycosyl hydrolase domain and a DDH domain, respectively. The recovered peptide sequences covered 86% of the glycosyl hydrolase domain amino acid sequence. Most bacterial glycosyl hydrolases have been widely studied for their biochemical properties and structural functions [17,18]. Thus, the glycosyl hydrolase domain-containing protein was regarded as a potential core component contributing to the antifungal effects of *P. polymyxa*.

### 2.5. Expression and Purification of 76kd Recombinant Protein

A recombinant expression plasmid carrying the complete coding sequence of the target 76 kDa protein was assembled for heterologous protein expression in *E. coli*. A full schematic workflow illustrating the gene cloning and vector construction procedures is attached in the Supplementary Materials (Figure S1). The successfully constructed recombinant plasmid pET32a(+)/76kd was chemically transformed into competent cells of *E. coli* BL21 (DE3) strain. The transformed bacterial strains were cultured at a constant temperature of 37 °C, and total cellular proteins were extracted from the induced bacterial pellets for subsequent SDS-PAGE electrophoretic detection to analyze the expression status of the target recombinant protein.

**Figure 3:**
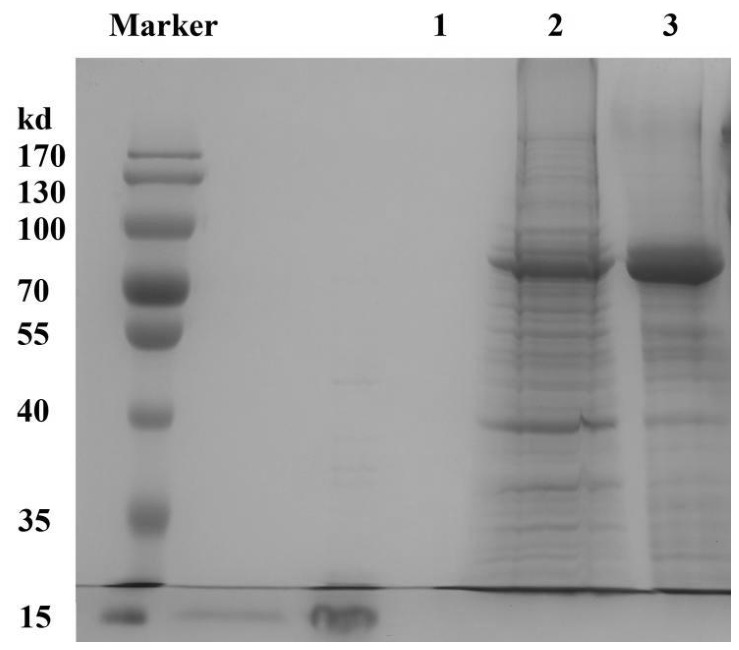
SDS-PAGE analysis of recombinant p76kd expressed in *E. coli* BL21 (DE3). Note: Lane 1 and Lane 2: the supernatant and pellet of the recombinant pET32a(+)/76kd cultures after induction in *E. coli* BL21, respectively; Lane 3: the expressed 76kd was purified by washing five times.

Electrophoretic band observation revealed that the *E. coli* BL21 (DE3) cells transformed with pET32a(+)/76kd yielded abundant recombinant protein p76kd with a molecular weight of approximately 76 kDa. The majority of the expressed p76kd protein was recovered within the insoluble precipitate fraction after cell lysis and centrifugation, as displayed in Lane 2 of Figure 3. The electrophoretic bands demonstrated that nearly all target p76kd protein accumulated in the form of insoluble inclusion bodies, corresponding to the protein sample loaded in Lane 3 of Figure 3.

Purification procedures were therefore carried out under denaturing buffer conditions to isolate the target inclusion bodies. The harvested inclusion body precipitates underwent five successive rounds of washing treatment using washing buffer supplemented with 2 M urea. After repeated washing steps, the purified inclusion body pellets were fully solubilized in denaturing buffer containing 8 M urea. SDS-PAGE electrophoresis was performed to compare protein purity between the unprocessed crude cell lysate and washed, solubilized inclusion body samples. The electrophoretic bands indicated that the purity of solubilized p76kd (Lane 3, Figure 3) was markedly higher relative to the crude total protein lysate (Lane 2, Figure 3).

**Figure 4:**
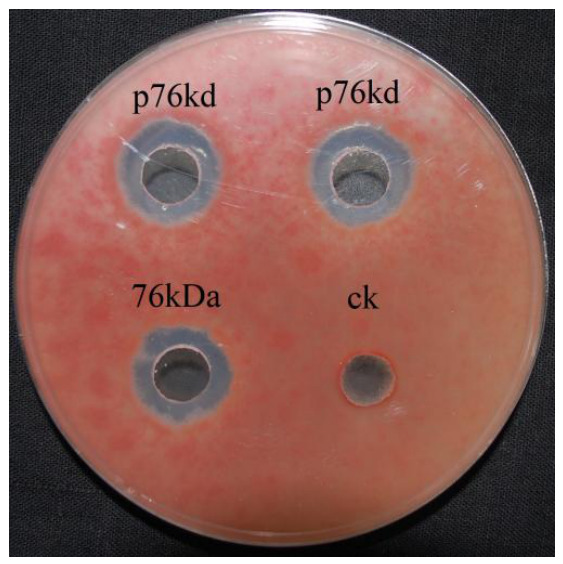
Antifungal activity comparison between renatured recombinant p76kd and native 76 kDa protein against *F. graminearum*.

The denatured, purified p76kd protein solution was subjected to refolding treatment via two combined methods: gradient urea dialysis and liquid dilution refolding. After completion of the renaturation process, antifungal activity testing was conducted on the refolded recombinant p76kd protein sample. The antifungal inhibition zone results presented in Figure 4 showed that the renatured recombinant p76kd protein displayed inhibitory activity levels comparable to the native 76 kDa antifungal protein extracted from the fermentation supernatant of *P. polymyxa*.

## 3. Discussion

Biological control of plant fungal diseases using beneficial bacteria has become a key research area in sustainable agriculture. *P*. *polymyxa* is widely recognized as a valuable biocontrol agent owing to its capacity to produce diverse antifungal metabolites, including lipopeptides, hydrolases, and low-molecular-weight proteins [5,9]. In this study, we isolated and identified a novel 76 kDa extracellular antifungal protein that exhibits broad-spectrum inhibitory activity against diverse *Fusarium* species. The extracellular localization of the antifungal component facilitates its purification and practical application. Similar observations have been reported in other *Bacillus* and *Paenibacillus* strains, where antimicrobial proteins are mainly secreted into the culture supernatant [14,19]. The excellent thermostability (40–90 °C) and pH stability (3.0–9.0) of the 76 kDa protein suggest high potential for industrial processing and field application, as many biocontrol products require tolerance to heat and extreme pH conditions during formulation and storage [20]. Sensitivity to proteases confirmed the proteinaceous nature of the active component, consistent with previous findings that bacteriocins and antifungal proteins from *P. polymyxa* are typically inactivated by proteolytic enzymes [12]. LC-MS/MS analysis identified a glycosyl hydrolase domain in the 76 kDa protein. Glycosyl hydrolases are key enzymes that degrade fungal cell wall polysaccharides such as chitin and β-1,3-glucans, thereby inhibiting hyphal growth and spore germination [8,21,22]. Recent studies have demonstrated that glycosyl hydrolases from biocontrol bacteria contribute significantly to the suppression of *Fusarium* and other phytopathogens [23,24,25]. LC-MS/MS analysis confirms this protein carries a conserved glycosyl hydrolase domain. Most previously reported antifungal proteins from *P. polymyxa* [16] range from 10–45 kDa and belong to chitinase or bacteriocin-like peptides. In contrast, our target protein is a 76 kDa glycosyl hydrolase with a unique dual-domain structure containing glycosyl hydrolase and DDH domains, which has rarely been documented in antagonistic *Paenibacillus* strains. Based on the previously documented cell-wall-degrading function of homologous glycosyl hydrolases from biocontrol bacteria, we speculate that the purified 76 kDa protein suppresses fungal growth via the hydrolytic degradation of fungal cell-wall polysaccharides; definitive enzymatic kinetic data of this unique protein will be supplemented in our subsequent enzymology research.

The purified 76 kDa protein showed antifungal activity against *F. graminearum*, the primary causal agent of FHB. Such activity is particularly advantageous for the development of robust biocontrol agents applicable to complex pathogen communities in the field. Furthermore, heterologous expression in *E. coli* generated a recombinant protein (p76kd) that, following renaturation, maintained antifungal activity equivalent to that of the native protein. Successful heterologous expression lays the foundation for large-scale production and further engineering of this antifungal protein [26,27]. Most reported antifungal compounds from *P. polymyxa* are lipopeptides such as fusaricidins [11,13]. In contrast, our study identifies a discrete antifungal protein with a defined glycosyl hydrolase domain, expanding the repertoire of biocontrol factors from *P. polymyxa*. These findings support the development of protein-based biopesticides as alternatives to chemical fungicides for FHB management.

Future research should focus on elucidating the precise hydrolytic activity and structural characteristics of the 76 kDa protein, evaluating its biocontrol efficacy in greenhouse and field trials, and optimizing fermentation and formulation processes for commercial application.

## 4. Conclusions

The dominant antifungal component from *P. polymyxa* resided in cell-free culture supernatant, confirming its extracellular localization. This bioactive component retained stable antifungal activity across 40–90 °C and pH 3.0–9.0 and was confirmed to be proteinaceous. A 76 kDa protein was purified sequentially using DEAE-52 anion-exchange and Sephadex G-75 gel-filtration chromatography, which displayed antifungal activity against *F. graminearum*. LC-MS/MS verified a conserved glycosyl hydrolase domain within the purified protein. The corresponding coding gene was cloned into pMD18-T before subcloning into the pET32a(+) expression vector, and recombinant constructs were validated via PCR, restriction digestion and DNA sequencing. The recombinant plasmid pET32a(+)/76kd was transformed and heterologously expressed in *E. coli* BL21(DE3), predominantly forming inclusion bodies. After refolding via gradient dialysis and dilution, the renatured recombinant p76kd possessed antifungal activity equivalent to the native 76 kDa protein. Collectively, *P. polymyxa* and its novel 76 kDa glycosyl hydrolase are promising biocontrol resources for managing wheat diseases triggered by *F. graminearum* and allied fungal pathogens.

## 5. Materials and Methods

### 5.1. Strain Vectors and Culture Conditions

*P. polymyxa* was kept in our laboratory [16]. *Fusarium graminearum* GZ3639 was provided by the Institute of Microbiology, Chinese Academy of Sciences (Beijing, China). *Escherichia coli* DH5α, *E. coli* BL21 (DE3), and the expression vector pET-32a(+) were preserved in our laboratory.

Bacterial strains were streaked onto Luria–Bertani (LB) agar (pH 7.0) from glycerol stocks stored at −70 °C and incubated at 30 °C for 24 h. A single colony was inoculated into 100 mL LB medium in 250 mL flasks and cultured at 37 °C and 150× *g* for 24 h to prepare the preculture (10^8^–10^9^ CFU mL^−1^). Then, 10% (*v*/*v*) preculture was inoculated into fresh LB medium and incubated under the same conditions for antifungal metabolite production [28].

### 5.2. Antifungal Activity Assay

Antifungal activity was determined using the agar well diffusion method [29]. Potato dextrose agar (PDA) plates were inoculated with a *F. graminearum* GZ3639 spore suspension (1 × 10^4^ spores mL^−1^). Samples (50 μL) were added into 6-mm wells. After incubation at 30 °C for 48 h, the diameter of the inhibition zone was measured. PBS (pH 6.8) was used as a negative control. All assays were performed in five replicates for quantitative reliability.

### 5.3. Localization of Antagonistic Components

The fermented culture medium was centrifuged in a refrigerated centrifuge (Kendro D-37250, Sorvall-Heraeus Scientific Instruments Co., Ltd., Hanau, Germany) at 6000× *g* for 15 min at 4 °C. The supernatant was collected as an extracellular fraction. The cells were washed twice with 0.9% NaCl and resuspended in 50 mM PBS (pH 6.8). Cell disruption was performed in an ice bath using a VCX500 sonicator (Sonics & Materials, Newtown, CT, USA) equipped with a microtip at 100 W with 10 pulses of 5 min each and 3 s intervals. After centrifugation at 12,000× *g* and 4 °C for 15 min, the supernatant was collected as the intracellular fraction and used for antagonistic activity assays.

### 5.4. Protein Precipitation and Sample Preparation

*P. polymyxa* was cultured after 12 h as described above. Ten milliliters of cell-free supernatant was precipitated with 60% ammonium sulfate at 4 °C overnight, as a gradual addition with slow stirring. The precipitate was dissolved, dialyzed and ultrafiltered (100 kDa, Amicon Ultra-15 Centrifugal Filter; Millipore, Billerica, MA, USA) and centrifugated at 8000× *g* for 15 min at 4 °C. Purification was performed using DEAE-52 anion exchange chromatography and Sephadex G-75 gel filtration chromatography. Active fractions were pooled and concentrated.

### 5.5. Biochemical Characterization of the Antifungal Component

Thermal inactivation was performed by incubating the protein samples at 40, 50, 60, 70, 80, and 90 °C for 30 min, and residual antifungal activity against *F. graminearum* GZ3639 was determined using the agar well diffusion method. For pH stability analysis, the supernatant pH was adjusted to a range of 2.0–10.0. After incubation at 37 °C for 1 h, residual activity was measured as described above. Sterile water adjusted to corresponding pH values served as the control. For protease sensitivity assays, the antagonist samples were adjusted to the optimal pH of each protease and treated at 37 °C for 1 h with 1 mg mL^−1^ proteinase K (pH 8.6), pepsin (pH 2.0), trypsin (pH 7.6), neutral protease (pH 7.0), or papain (pH 5.6; all from Sigma, St. Louis, MO, USA). Residual activity was determined using the agar well diffusion assay, and untreated samples were used as the control [30].

### 5.6. Purification of Antifungal Component

The cell-free supernatant was collected after centrifugation at 8000× *g* for 15 min. The antifungal component was precipitated overnight with 60% ammonium sulfate at 4 °C, followed by gentle stirring for an additional 1 h. The precipitate was dissolved in 50 mM PBS (pH 6.8), dialyzed against the same buffer using a cellulose membrane, and filtered through a 0.22 μm sterile filter (Membrana, Wuppertal, Germany) to obtain a clear solution. The sample was then loaded onto a DEAE-52 anion-exchange column (1.6 cm × 20 cm) pre-equilibrated with 100 mL of 50 mM PBS (pH 6.8). Elution was performed with a linear NaCl gradient (0–1.0 mol L^−1^) at a flow rate of 1.0 mL min^−1^, and 5 mL fractions were collected and monitored at 280 nm. Active fractions were combined, dialyzed, concentrated, and further loaded onto a Sephadex G-75 gel filtration column (1.0 cm × 70 cm) pre-equilibrated with 50 mM PBS (pH 6.8). Elution was carried out using the same buffer at a flow rate of 0.1 mL min^−1^. Active fractions were pooled and concentrated for subsequent analysis. SDS-PAGE was performed to assess protein purity and estimate molecular weight, using a 5% stacking gel and 12% separating gel. A 20 μL aliquot of the sample was mixed with an equal volume of 2 × loading buffer and heated at 100 °C for 3 min. A protein molecular weight marker (10.0–170.0 kDa; Sigma, St. Louis, MO, USA) was used for reference. Electrophoresis was conducted at 80 V for the stacking gel and 130 V for the separating gel [16].

### 5.7. Mass Spectrometry

The molecular mass and structural characteristics of the purified antifungal protein were determined using reversed-phase capillary LC-MS [31,32]. For further structural identification, the purified protein was analyzed using matrix-assisted laser desorption/ionization time-of-flight tandem mass spectrometry (MALDI-TOF-TOF; UltraflexIII, Bruker Daltonics, Billerica, MA, USA). Peptide mass fingerprinting data were processed using the Mascot sequence matching software (Matrix Science, London, UK; http://www.matrixscience.com, accessed on 21 June 2025). Protein identification was evaluated based on Mascot score, number of matched peptides, theoretical molecular mass, and sequence coverage.

### 5.8. Prokaryotic Expression and Purification of Recombinant p76kd

The amplified 76 kd gene was directionally cloned into the pMD18-T vector as previously researched [16]. Following sequence verification, the insert was released by BamH I/Xho I digestion, recovered using a TaKaRa gel purification kit, and directionally ligated into the similarly digested expression vector pET32a(+), yielding the recombinant plasmid pET32a(+)/76 kd. After ligation and transformation, positive recombinant plasmids were extracted and sequenced using the promoter forward primer and terminator reverse primer matching the pET32a(+) vector backbone. The resulting raw sequencing reads were aligned against the target gene’s theoretical coding sequence to confirm 100% sequence identity without mutation. Positive clones were confirmed by PCR, restriction enzyme digestion, and DNA sequencing (TaKaRa). The confirmed recombinant plasmid was transformed into *E. coli* BL21 (DE3) competent cells. Protein expression was induced using isopropyl-β-D-thiogalactopyranoside (IPTG). Cells were harvested by centrifugation, lysed in 5×SDS sample buffer (0.1 M Tris-HCl, 4% SDS, 0.2% bromophenol blue, 20% glycerol, 0.1 M DTT, pH 6.8), and analyzed by SDS-PAGE. Uninduced cultures and cultures harboring empty pET32a(+) vectors were analyzed in parallel as controls.

Recombinant p76kd protein was purified under denaturing conditions via repeated washing. Induced cells were harvested by centrifugation at 8000× *g* for 10 min and resuspended in 20 mM Tris buffer (pH 8.0) containing 0.1 mg mL^−1^ lysozyme, followed by incubation at −20 °C overnight. Cell lysis was performed by sonication on ice for 5 min at 30% amplitude with 30 s pulses. After centrifugation at 8000× *g* for 10 min, the supernatant (soluble fraction) and pellet (insoluble fraction) were collected separately for SDS-PAGE analysis. Results showed that recombinant p76kd was primarily produced as inclusion bodies (IBs). The pellets were resuspended in 20 mL washing buffer (2 M urea, 50 mM Tris-HCl, 1 mM EDTA, 150 mM NaCl, 0.1% Triton X-100, pH 8.0), mixed gently with constant stirring for 10 min, and centrifuged at 8000× *g* for 10 min at 4 °C. This washing step was repeated five times to remove impurities. The final pellet was collected by centrifugation at 8000× *g* for 10 min at 4 °C and resuspended in denaturing buffer (8 M urea, 10 mM PBS, 50 mM Tris-HCl, 50 mM NaCl, 10% glycerol, pH 8.0). The purity of p76kd was tested by SDS-PAGE, and the activity was tested using an antifungal assay [26,27].

### 5.9. Statistical Analysis

All data are presented as mean ± standard deviation from five independent biological replicates. Statistical analyses were conducted via one-way analysis of variance (ANOVA) followed by Tukey’s post hoc test using SPSS 18.0 software, with significance set at α = 0.05.

## Figures and Tables

**Table 1:** Thermal, pH and protease stability of the antifungal component from *P. polymyxa*.

Factors	Source	Diameter of Inhibition Circle (mm) (Mean ± Standard Deviation)
Thermal	40	16.82 ± 1.32 ^a^
	50	15.13 ± 1.41 ^a^
	60	14.57 ± 1.05 ^b^
	70	12.84 ± 1.67 ^c^
	80	7.87 ± 0.45 ^d^
	90	6.13 ± 0.36 ^d^
pH	2.0	0
	3.0	2.35 ± 0.08 ^e^
	4.0	6.12 ± 0.15 ^d^
	5.0	10.65 ± 1.84 ^c^
	6.0	14.25 ± 1.66 ^b^
	7.0	16.12 ± 1.58 ^a^
	8.0	13.89 ± 1.34 ^b^
	9.0	11.25 ± 1.02 ^c^
	10.0	0
Protease	Proteinase K	2.65 ± 0.03 ^c^
	Pepsin	15.68 ± 1.47 ^a^
	Trypsin	3.41 ± 0.16 ^c^
	Neutral protease	5.12 ± 0.24 ^b^
	Papain	14.37 ± 1.12 ^a^
CK		16.85 ± 1.67 ^a^

Note: Values are presented as mean ± standard deviation. Means labelled with identical lowercase letters within the same column show no statistically significant difference (*p* > 0.05), while means marked with distinct lowercase letters are significantly different (*p* < 0.05), as determined by one-way analysis of variance (ANOVA) followed by Tukey’s post hoc test.

**Table 2:** Antifungal activity of chromatographic fractions separated during protein purification.

Fractions	Antifungal Diameter/mm (Mean ± Standard Deviation)
D1	-
D2	-
D3	7.65 ± 0.27 ^b^
D4	9.77 ± 0.18 ^a^
D5	-
S1	-
S2	9.04 ± 0.15 ^a^
S3	-

Note: Values are presented as mean ± standard deviation. Means labelled with identical lowercase letters within the same column show no statistically significant difference (*p* > 0.05), while means marked with distinct lowercase letters are significantly different (*p* < 0.05), as determined by one-way analysis of variance (ANOVA) followed by Tukey’s post-hoc test.

## Data Availability

The original contributions presented in this study are included in the article/Supplementary Materials. Further inquiries can be directed to the corresponding author.

## Supplementary Materials

The following supporting information can be downloaded at: https://www.mdpi.com/article/10.3390/toxins18070318/s1. Figure S1. The cloning strategy for constructing the 76 kDa recombinant plasmids.
