# Supplementary material for: Molecular Identification and Recombinant Expression of a Novel Antifungal Protein from Wheat-Associated Paenibacillus polymyxa
Source: Toxins (Basel). 2026 Jul 22;18(7):318. doi: 10.3390/toxins18070318 (PMC13416985; doi:10.3390/toxins18070318)
Supplement: Supplementary file 1 [file toxins-18-00318-s001.zip › toxins-4449475-supplementary.pdf]

Supplementary Materials: Molecular Identification and Recombinant Expression of a Novel Antifungal Protein from Wheat-Associated *Paenibacillus polymyxa*

Xiaohong Ge, Zhikun Chen, Haoyuan Guo and Junjian Ran

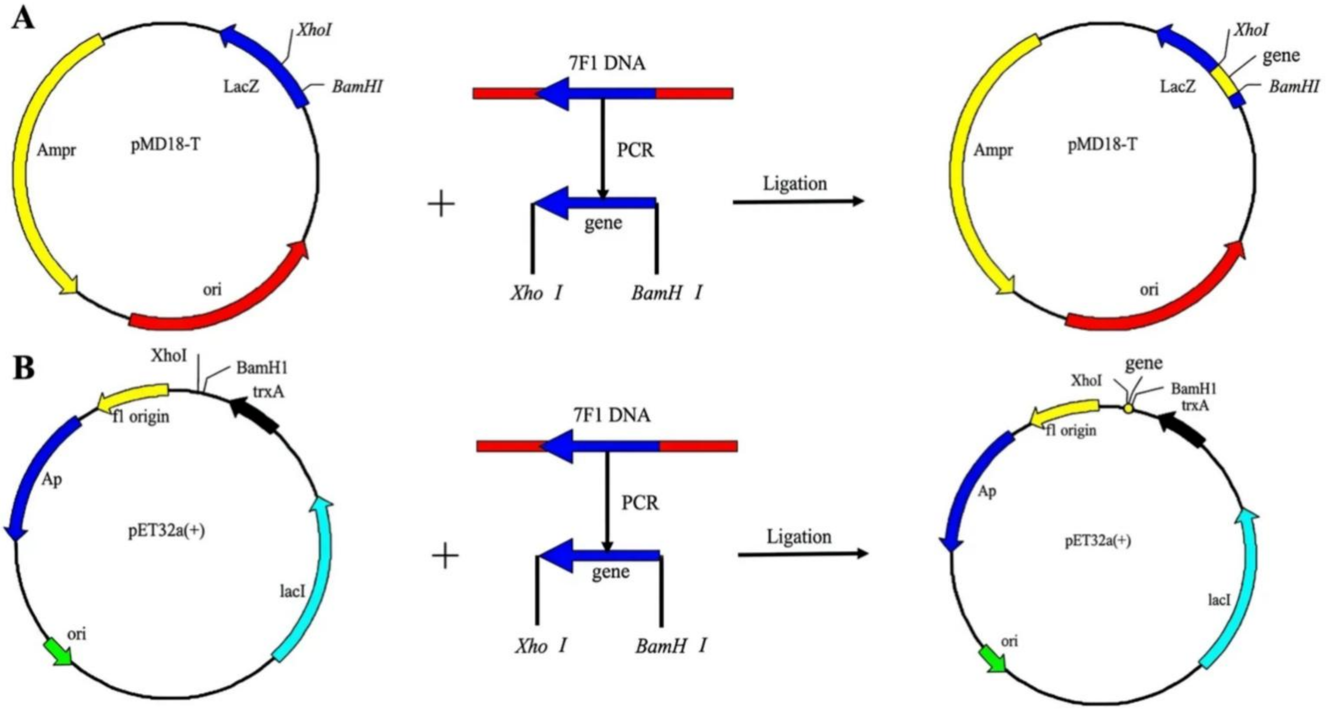

Figure S1. The cloning strategy for constructing the 76 kDa recombinant plasmids.
